# Knockdown of GTF2E2 inhibits the growth and progression of lung adenocarcinoma via RPS4X in vitro and in vivo

**DOI:** 10.1186/s12935-021-01878-z

**Published:** 2021-03-23

**Authors:** Guoshu Bi, Donglin Zhu, Yunyi Bian, Yiwei Huang, Cheng Zhan, Yong Yang, Qun Wang

**Affiliations:** 1 Department of Thoracic Surgery, Zhongshan Hospital, Fudan University, No. 180 Fenglin Road, Xuhui District, Shanghai, 200032 China; 2 Department of Thoracic Surgery, The Affiliated Suzhou Hospital of Nanjing Medical University, Suzhou, 215000 Jiangsu Province China

## Abstract

**Background:**

Lung adenocarcinoma (LUAD) is one of the most common malignancies worldwide. However, the molecular mechanism of LUAD tumorigenesis and development remains unclear. The purpose of this study was to comprehensively illustrate the role of GTF2E2 in the growth and progression of LUAD.

**Methods and materials:**

We obtained the mRNA expression data from The Cancer Genome Atlas, Gene Expression Omnibus database, and our institution. Systematic bioinformatical analyses were performed to investigate the expression and prognostic value of GTF2E2 in LUAD. The results were validated by immunohistochemistry and qPCR. The effect of knocking down GTF2E2 using two short hairpin RNAs was investigated by in vitro and in vivo assays. Subsequently, shotgun liquid chromatography coupled with tandem mass spectrometry (LC–MS/MS) analyses were applied to identified potential GTF2E2 interacting proteins, and the downstream molecular mechanisms of GTF2E2-signaling were further explored by a series of cellular functional assays.

**Results:**

We found that GTF2E2 expression was significantly increased in LUAD tissue compared with adjacent normal tissue and was negatively associated with patients’ overall survival. Besides, we demonstrated that GTF2E2 knockdown inhibited LUAD cell proliferation, migration, invasion, and promote apoptosis in vitro, as well as attenuated tumor growth in vivo. Results from LC–MS/MS suggested that RPS4X might physically interact with GTF2E2 and mediated GTF2E2’s regulatory effect on LUAD development through the mTOR pathway.

**Conclusion:**

Our findings indicate that GTF2E2 promotes LUAD development by activating RPS4X. Therefore, GTF2E2 might serve as a promising biomarker for the diagnosis and prognosis of LUAD patients, thus shedding light on the precise and personalized therapy for LUAD in the future.

**Supplementary Information:**

The online version contains supplementary material available at 10.1186/s12935-021-01878-z.

## Introduction

Lung cancer is the leading cause of cancer incidence and mortality worldwide, with 2.1 million new diagnoses and 1.8 million deaths estimated in 2018 [1], and lung adenocarcinoma (LUAD) is currently the most common subtype of non-small cell lung cancer, accounting for more than 40% of total cases [2]. According to the National Comprehensive Cancer Network guidelines, surgery combined with (neo)adjuvant chemotherapy or radiotherapy remains the standard procedure for treating lung malignancies. Simultaneously, the application of immunotherapy and target therapy has recently delivered unprecedented success to extend patients’ overall survival [3–5]. However, lung cancer’s age-standardized 5-year survival remains pretty low (10–20%) in most countries [6, 7]. Therefore, it is critical to explore new oncological biomarkers and underlying molecular mechanisms involved in the progression of LUAD.

Transcription initiation factor IIE subunit beta, also known as GTF2E2, plays a vital role in the initiation of RNA transcription by recruiting TFIIH to the initiation complex and stimulating the RNA polymerase II C-terminal domain kinase and DNA-dependent ATPase activities of TFIIH [8–10]. GTF2E2 serves as a limiting member for the formation of its protein complex (GTF2E1–GTF2E2) since this process is impacted by post-transcriptional regulation like protein-degradation [11]. Moreover, Yang et al.’s bioinformatic study suggested that GTF2E2 promotes the development of glioblastoma by upregulating the expression of the cell division cycle 20 (CDC20) [12]. Besides, GTF2E2 mutation indicates defective DNA repair-independent transcription and tissue-specific dysfunction [13]. However, the role of GTF2E2 plays in tumors remains unclear.

Therefore, in the present study, we systematically examined GTF2E2’s potential functions in tumorigenesis and metastasis of LUAD and explored the downstream molecules probably interacting with it. Together, our findings indicate that GTF2E2 may serve as a prognostic factor and potential therapeutic target for LUAD.

## Methods and materials

### Patients and LUAD specimens

Human surgical specimens, including both tumor and adjacent noncancerous tissue, were obtained from 153 patients with stage I LUAD who underwent lobectomy and systematic lymph node resection at the Department of Thoracic Surgery, Zhongshan Hospital, Fudan University from 2016 to 2017. All patients provided written informed consent to conduct genomic studies in accordance with the ethical principles of the Declaration of Helsinki, the International Conference on Harmonization Guidelines for Good Clinical Practice. All pulmonary resections were performed by experienced thoracic surgeons in our institution, and resected tumors and lymph node specimens were all labeled in the operating theater and reviewed by at least two qualified pathologists to confirm the diagnosis of LUAD through hematoxylin and eosin-stained sections and immunochemical analysis. RNA sequencing for the 34 pairs of stage I tumor samples was performed using Illumina Hiseq 2500 and BGI-500RNAseq platforms, and immunohistochemistry for the remaining 119 couples was performed as follows. The study was approved by the ethical committees of Zhongshan Hospital (No. 201986122).

### Bioinformatic analysis

Level 4 gene expression data of LUAD patients (FPKM normalized) and corresponding clinical information of The Cancer Genome Atlas (TCGA) were downloaded from the UCSC Xena browser (GDC hub: https://gdc.xenahubs.net). We removed patients whose clinical outcome information were vague or absent, including survival time and vital status. For Gene Expression Omnibus (GEO) database, microarray data in GSE30219 datasets and corresponding clinical information were obtained from https://www.ncbi.nlm.nih.gov/geo. The probe sets of Affymetrix Human Genome U133 Plus 2.0 were annotated to gene names based on the annotation platforms GPL570. We also retrospectively selected 34 patients with stage I LUAD who underwent lobectomy and systematic lymph node resection at our institution from 2016 to 2017 and performed RNA sequencing for all tumor samples. Besides, single-cell sequencing analysis was also performed. The detailed procedure was as described in our previous study [14, 15].

### Immunohistochemistry

Tissue specimens were obtained from 119 patients who received surgery between February 2014 and December 2018 and were histologically diagnosed with LUAD. The paraffin-embedded tissues were dewaxed, rehydrated, and stained using the GTVisionTM + Detection System/Mo&Rb Immunohistochemistry kit according to the manufacturer’s protocol (GK500710, GeneTech, Shanghai, China). Anti-GTF2E2 antibody (1:100, abs118610, Absin Bioscience Inc, Shanghai, China) was used in this part. The detailed procedure was as described in our previous study [16].

### RNA extraction and quantitative real-time polymerase chain reaction (qRT-PCR)

Trizol reagent was used to extract total RNA from frozen tissues and tumor cells enrolled in our study. Then the PrimeScript RT Reagent Kit (TaKaRa, Tokyo, Japan) was used to synthesize the cDNA template, and SYBR Premix Ex Taq was used to perform qRT-PCR analysis according to the manufacturer’s protocol. All reactions were analyzed in an Applied Biosystems system 7500 (Thermo Fisher Scientific, Waltham, MA, USA). Relative quantification of mRNA was calculated using the 2-ΔΔCT method using GAPDH as an endogenous calibrator. All primers were synthesized by Sangon Biotech (Shanghai, China), and their sequences are provided in Additional file 1: Table S1. Triplicate experiments were performed in each sample.

### Cell lines and lentivirus transfection

LUAD cell lines (A549 and H1299) were purchased from the Chinese Academy of Science Cell Bank. Cells were cultured in high-glucose Dulbecco’s Modified Eagle’s Medium (DMEM, Hyclone, Logan, UT, USA) supplemented with 10% fetal bovine serum (Every Green, Zhejiang, China) and 100 U/ml penicillin/streptomycin/amphotericin B (Sangon Biotech) in a humidified 5% CO_2_ atmosphere at 37 ℃. Two different short hairpin RNAs (shRNAs) targeting GTF2E2 and corresponding control were designed and cloned into lentiviral vectors with GFP fluorescence by Genechem Co., Ltd, Shanghai, China. Target sequences of the shRNAs and negative-controls are provided in Additional file 1: Table S1. A549 and H1299 cells were transfected with the lentivirus using polybrene (Life Technologies, Thermo Fisher Scientific) according to the manufacturer’s protocol. In addition, lentivirus containing full-length GTF2E2 cDNAs was also synthesized and transfected into A549 and H1299 to construct GTF2E2 overexpressing cell lines.

### Western blot analyses

Western blotting was performed according to standard procedures as previously described [16]. Proteins were extracted from frozen tissues and cells using RIPA buffer (Beyotime, Shanghai, China) with protease and phosphatase inhibitor cocktail (Beyotime) and quantified using an Enhanced BCA Protein Assay Kit (Beyotime). Proteins were then separated by SDS-PAGE and transferred onto polyvinylidene fluoride membranes (Merck-Millipore, Burlington, MA, USA) (Constant current 0.32 A, 90 min). The membranes were blocked with 5% nonfat milk for 1 h and then incubated with specific primary antibodies for 12 h at 4 °C. After washing the membranes three times with Tris-buffered saline-Tween (TBST) solution, the secondary antibody dilutions were incubated on the membranes at room temperature for 1 h. Finally, the protein bands were visualized by Moon Chemiluminescence Reagent kit (Beyotime). In this study, the following antibodies were used: Anti-GTF2E2 (1:2000, HPA025065, Sigma-Aldrich, St. Louis, Missouri, USA), anti-GAPDH (1:2000, sc-32233, Santa Cruz Biotechnology, Texas, USA), anti-flag (for CO-IP, A2220, Sigma-Aldrich), anti-ENO1 (1:500, 11204-1-AP, Proteintech, Wuhan, Hubei Province, China), anti-HSPB1 (1:1000, #2402, CST, Danvers, Massachusetts, USA), anti-NSUN1 (1:3000, sc-398884, Santa Cruz Biotechnology), anti-PARP1 (1:5000, 66520-1-Ig, Proteintech), anti-RPL31 (1:1000, SAB1307227, Sigma-Aldrich), anti-RPS4X (1:500, ab138065, Abcam, Cambridge, UK), anti-phospho-S6K1 (1:1000, abs130855, Absin), anti-phospho-eIF4E (1:3000, abs13328, Absin), horseradish peroxidase (HRP)-labeled goat anti-rabbit IgG (H + L) (1:2000, #7076, CST), and HRP-labeled goat anti-mouse IgG (H + L) (1:2000, #7074, CST).

### Cell proliferation analyses

A total of 1500 cells at logarithmic growth phase were seeded into black 96-well plates (Life Science, Oneonta, NY, USA) at 100 µL of cell suspension per well. Following incubation for 0, 24, 48, 72, 96, and 120 h at 37 °C, cell proliferation was measured according to corresponding fluorescence intensity using Celigo cytometer (Cyntellect Inc, San Diego, CA, USA), which is equipped with a 4-megapixel CCD camera with an F-theta scan lens.

### Colony formation assays

Cells were seeded in triplicate into 6-well plates at a density of 500 cells for A549 and 2000 cells for H1299 per well at logarithmic growth phase. After being cultured in complete culture medium at 37 ℃ for 14 days, the cells were fixed with 4% methanol for 30 min and stained with 1% purple crystal. ImageJ software (National Institute of Health, Bethesda, MD, USA) was used to count the colonies’ number with a diameter larger than 0.2 mm.

### Wound healing assays

Cells were seeded in triplicate into 96-well plate at a density of 50,000 cells per well at logarithmic growth phase and cultured in 37 ℃. When the cells reached a confluence > 90%, the 96-wounding replicator (V&P Scientific, San Diego, USA) was used to generate scratches in each well. Then we washed the cells 2 times using serum-free DMEM to remove cell debris and cultured them with low-serum DMEM. Wound photographs were scanned and analyzed at specific times with Celigo cytometer.

### Cell viability assays

Cells were seeded in triplicate into 96-well plate at a density of 2000 cells per well at logarithmic growth phase. Following incubation for 0, 24, 48, 72, 96, and 120 h at 37 °C, cell viability was determined by MTT assay (Gen-View Scientific Inc, USA) according to the manufacturer’s protocol.

### Cell migration and invasion assays

The migratory and invasive capacities of A549 and H1299 cells were detected using 24-well transwell plates (Corning, NY, USA) with a pore size of 8.0 μm. For migration assays, cells were seeded at a density of 40,000 cells per well at logarithmic growth phase in 100 μl DMEM in the upper chamber. Then we added 500 μl of DMEM containing 30% FBS to the lower chamber. According to the manufacturer’s protocol, the inset was coated with Matrigel (Corning) for invasion assays. Then we seeded 80,000 cells in 500 μl DMEM per well in the upper chamber and added 750 μl DMEM containing 30% FBS to the lower one. After 24 h-incubation of the cells, we removed the cells remaining on the upper surface of the membrane and then used 4% paraformaldehyde to fix the cells that had migrated or invaded to the lower surface of the membrane. Then crystal violet was applied for staining the fixed cells for 15 min. Four 100 × and nine 200 × microscopic fields were randomly captured to count the stained cells using an IX71 inverted microscope (Olympus Corporation, Tokyo, Japan).

### Cell apoptosis assays

The apoptosis rate of A549 and H1299 cells were measured using the Annexin V Apoptosis Detection Kit FITC (eBioscience, San Diego, CA, USA). Cells were washed in PBS, resuspended in 1 × Binding buffer, incubated in fluorochrome-conjugated Annexin V, and analyzed by flow cytometry (BD Accuri C6 Plus, BD Biosciences, Franklin Lakes, NJ, USA) as suggested in the manufacturer’s protocol.

### Subcutaneous tumor formation

Female BACB/c nude mice (4-weeks-old) were purchased from GemPharmatech Co., Ltd, (Jiangsu, China) and raised in laminar flow cabinets under standard pathogen-free conditions. For the subcutaneous mouse model, 1 × 10^6^ A549 cells (Group 1, sh-control; Group 2, sh1-GTF2E2, Group 3, sh2-GTF2E2) were respectively subcutaneously injected into the right flanks of the mice (8/group). One month later, the tumors were measured using vernier caliper twice a week. Tumor volume was calculated as π/6 × length × width × width. Six weeks after primary injection, all mice were photographed by in vivo imaging system (Perkin Elmer, USA) and then sacrificed. The tumors were isolated and weighed for further analyses.

### Co-immunoprecipitation and mass spectrometry (Co-IP/MS)

Lentivirus containing flag-GTF2E2 cDNAs or negative control vector were synthesized and transfected into 293T cells for CO-IP analysis. Proteins were extracted from 293T cells using RIPA buffer (Beyotime) with protease and phosphatase inhibitor cocktail (Beyotime) and quantified using an Enhanced BCA Protein Assay Kit (Beyotime). After centrifugation at 10,000*g* for 30 min at 4 °C, the supernatant was collected. Anti-flag beads were then added into the protein lysates and incubated on a rotating wheel at 4 °C overnight. After incubation, the immunocomplex samples were collected by centrifugation at 8000 RPM for 5 min at 4 °C and washed five times with TBS buffer. Finally, the samples were boiled in SDS-PAGE buffer (Beyotime) and ready for the following analyses, including SDS-PAGE and western blot.

For MS analysis, the detailed procedure was previously described by Li et al. [17]. In brief, firstly, the gels were cut into small pieces and decolorized by 1 ml 100 mM NH_4_HCO_3,_ and then dried with 30% acetonitrile. Trypsin (Beyotime) at a concentration of 2.5 μg/ml in 40 mM NH_4_HCO_3_ buffer was added, and digestion was carried out overnight at 37 °C. The peptides were extracted from the gel, pooled, lyophilized, and stored at − 80 °C until use. Afterward, the extracted peptides were detected through nano LC–MS/MS on a Q Exactive mass spectrometer, and the results were analyzed by Mascot (Matrix Science, London, UK; version 2.3.0).

### Statistical analysis

All statistical analyses were conducted using Graphpad Prism software (7.0) and R software (Version 3.5.3; R Foundation for Statistical Computing, Vienna, Austria). Student’s t-test and the Wilcoxon test were performed to compare continuous variables between two groups, while one-way ANOVA for the comparison among multiple groups. Kaplan–Meier survival curves visualized by *ggplot2* package and log-rank tests were used to compare overall survival (OS) between different populations. The p values were all two-sided, and p values < 0.05 were considered significant.

## Results

### GTF2E2 is upregulated in LUAD and is associated with poor clinical outcome

**Fig. 1:**
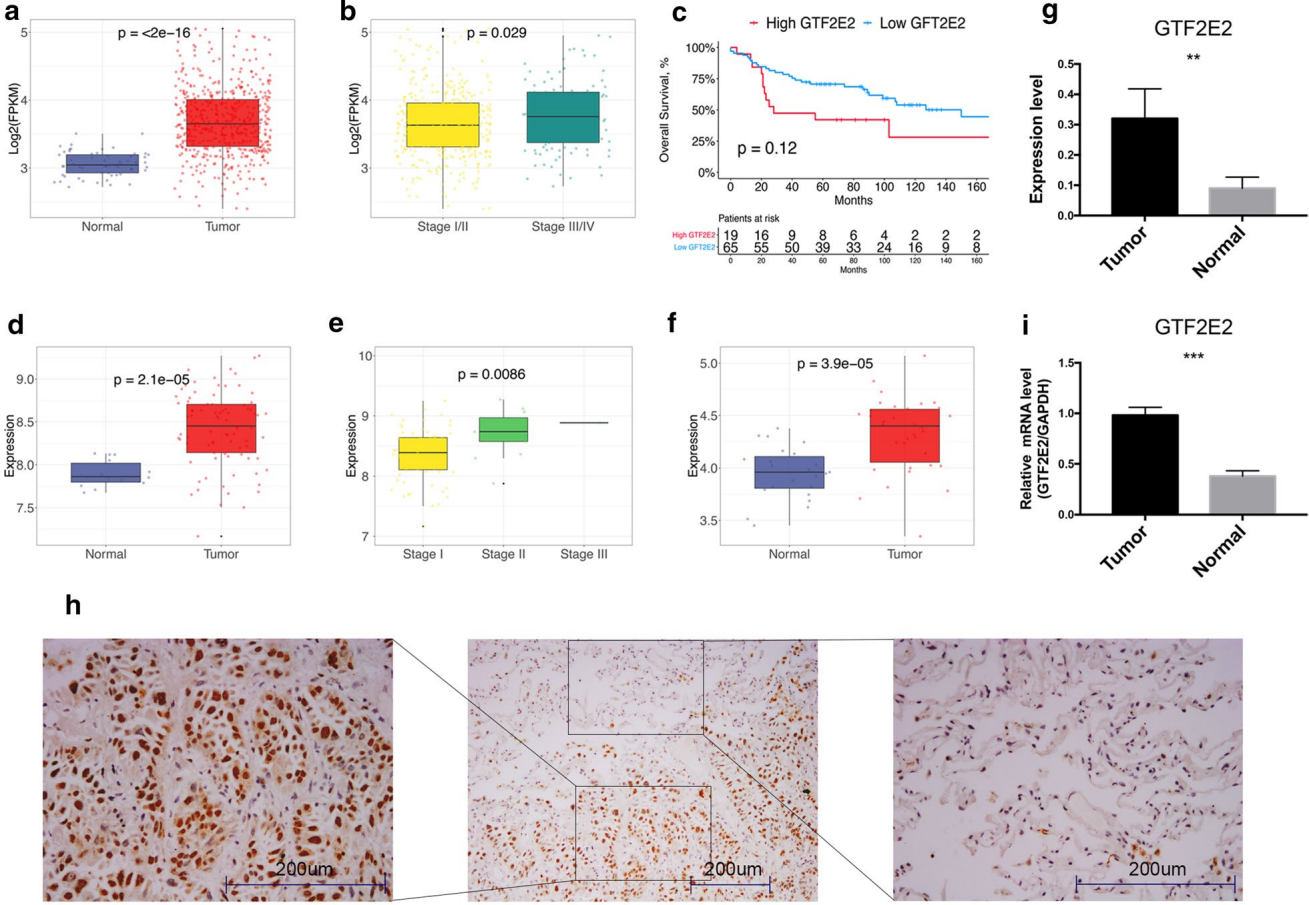
**a**, **d**, **f** Boxplots displaying the expression of GTF2E2 in lung adenocarcinoma and adjacent normal tissue based on the RNA sequencing or microarray data from TCGA (**a**), GEO (**b**), and our institution (**f**). Within each group, the scattered dots represent the value of each individual patient. The lines in the boxes represent the median value. The bottom and top of the boxes are the 25th and 75th percentiles (interquartile range). The whiskers encompass 1.5 times the interquartile range. The statistical difference of two scores was compared through the Wilcoxon test. **b**, **e** Boxplots displaying the expression of GTF2E2 in early and advanced lung adenocarcinoma tissue based on the RNA sequencing data from TCGA (**b**), and GEO (**e**). **c** Kaplan–Meier curves of overall survival stratified by GTF2E2 level in TCGA. **g**, **i** Quantitative RT-PCR and single-cell sequencing analyses of the expression of GTF2E2 in surgical resected lung adenocarcinoma and adjacent normal tissue. **h** Representative IHC staining images indicating the upregulation of GTF2E2 in lung adenocarcinoma. **p < 0.01; ***p < 0.001

To explore the potential biological function of GTF2E2 in LUAD, we first examined its expression level in tumor and adjacent normal tissues through multiple bioinformatical and experimental approaches. Based on RNA-sequencing data in TCGA database (Fig. 1a), the mRNA level of GTF2E2 was found to be significantly higher in tumor samples than in normal tissue (p < 0.001). Moreover, we noticed the upregulation of GTF2E2 in advanced-stage tumors (stage III/IV) in comparison with those in the early-stage (Fig. 1b). Furthermore, we performed survival analysis to investigate the prognostic value of GTF2E2 and classified LUAD patients into high or low GTF2E2 groups based on the optimal cutoff value identified by *survminer* package. As shown in Fig. 1c, patients with higher GTF2E2 were associated with significantly worse overall survival (log-rank p = 0.033). To further validate our findings, similar analyses were conducted in the microarray data from GEO database (GSE30219), which demonstrated the same results (Fig. 1d–e). We also obtained the mRNA sequencing data of 34 stage I LUAD patients who have received lobectomy in our institution and observed the increased GTF2E2 expression in tumor tissues (Fig. 1f). However, because of the limited sample size and follow-up time, we failed to note the significant prognostic difference between patients with a high or low level of GTF2E2 in these two independent datasets. A future large-scale study is warranted to further validate our results. Considering the potential heterogeneity, especially the confounding effect of non-tumor cells like immune cells and fibroblast infiltrating in bulk tumor tissues, we performed single-cell sequencing analysis to exhibit further the differential expression of GTF2E2 in isolated tumor and nontumor cells (Fig. 1g). Moreover, as for experimental evidence, the Overexpression of GTF2E2, including both mRNA and protein, in resected tumors in comparison with adjacent normal tissue, was confirmed by immunohistochemistry and qPCR. (Fig. 1h–i). Together, these results suggest that GTF2E2 may serve as an oncogene of LUAD.

### Knockdown of GTF2E2 inhibits proliferation and metastasis in LUAD cells

**Fig. 2:**
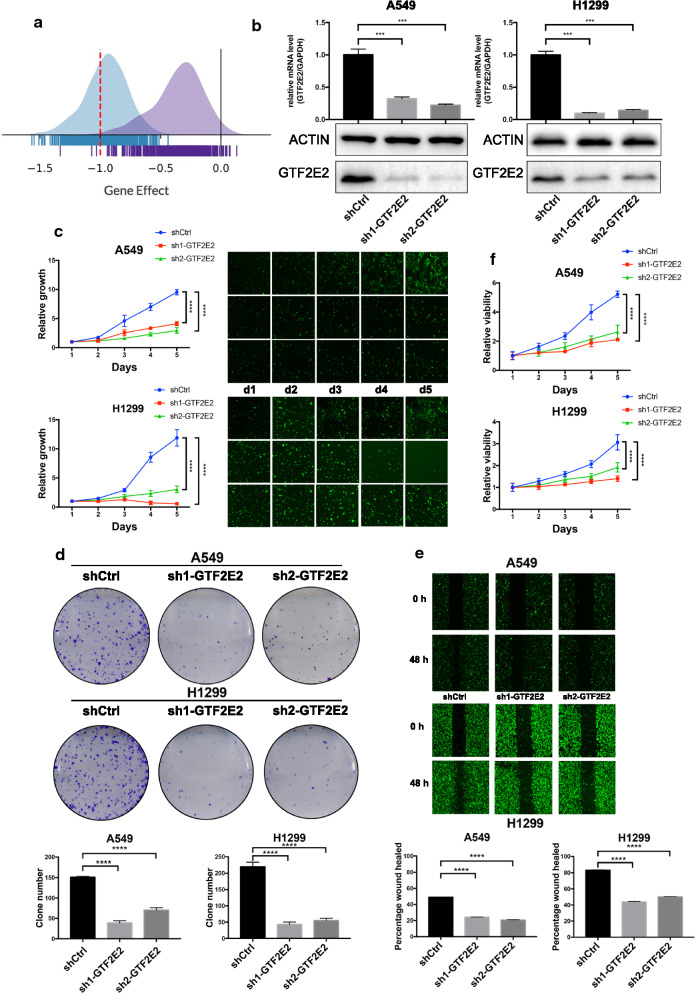
**a** The estimated value of gene dependency of GTF2E2 in Depmap database. **b** Quantitative RT-PCR and western blotting analyses verifying the GTF2E2 knockdown efficiency in A549 and H1299 cells. **c**–**f** The effects of GTF2E2 knockdown on cell proliferation (**c**), colony formation (**d**), migration (**e**), and viability (**f**) in A549 and H1299 cells. ***p < 0.001, ****p < 0.0001

**Fig. 3:**
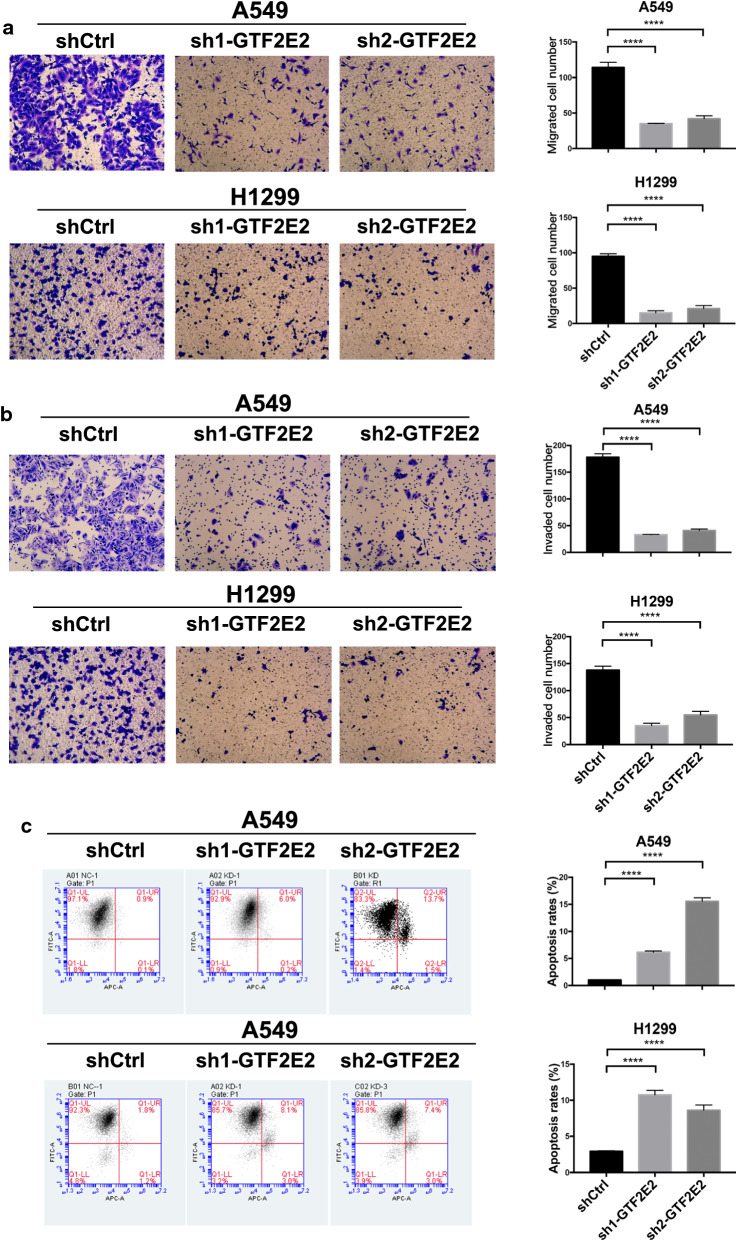
The effects of GTF2E2 knockdown on cell invasiveness and apoptosis exhibited by transwell (**a**), invasion assays (**b**), and flow cytometry (**c**) in A549 and H1299 cells. ****p < 0.0001

Based on the high-throughput data obtained from Crispr/Cas9 screening system in the DepMap database (https://depmap.org/), perturbation of GTF2E2 by RNAi in different tumors cell lines caused growth inhibition, indicating that it appears to serve as an oncogene in several tumor types (Fig. 2a). To further validate this finding and investigate whether GTF2E2 is a functional gene in tumorigenesis and development of LUAD, we designed two different shRNAs targeting GTF2E2 to avoid off-target effects and transfected them into two LUAD cell lines (A549 and H1299 cell lines) using lentiviral vectors. The stable knockdown efficiencies by shRNA were verified by comparing with the control cells at both mRNA and protein levels (Fig. 2b). Results from cell counting and clone formation assay demonstrated that the proliferation and tumorigenesis ability of GTF2E2 knockdown A549 and H1299 were significantly decreased compared to the control cells (Fig. 2c, d). In addition, wound healing assays and MTT assays showed that knocking down GTF2E2 significantly impeded cell migration abilities and viabilities (Fig. 2e, f). We also performed transwell assays and observed that the migratory and invasive capacities were greatly hindered by knocking down GTF2E2 (Fig. 3a, b). Moreover, as shown in Fig. 3c, in GTF2E2 knockdown A549 and H1299, the apoptosis rate, as measured by flow cytometry, was significantly enhanced than that in corresponding control cells (all p < 0.0001).

**Fig. 4:**
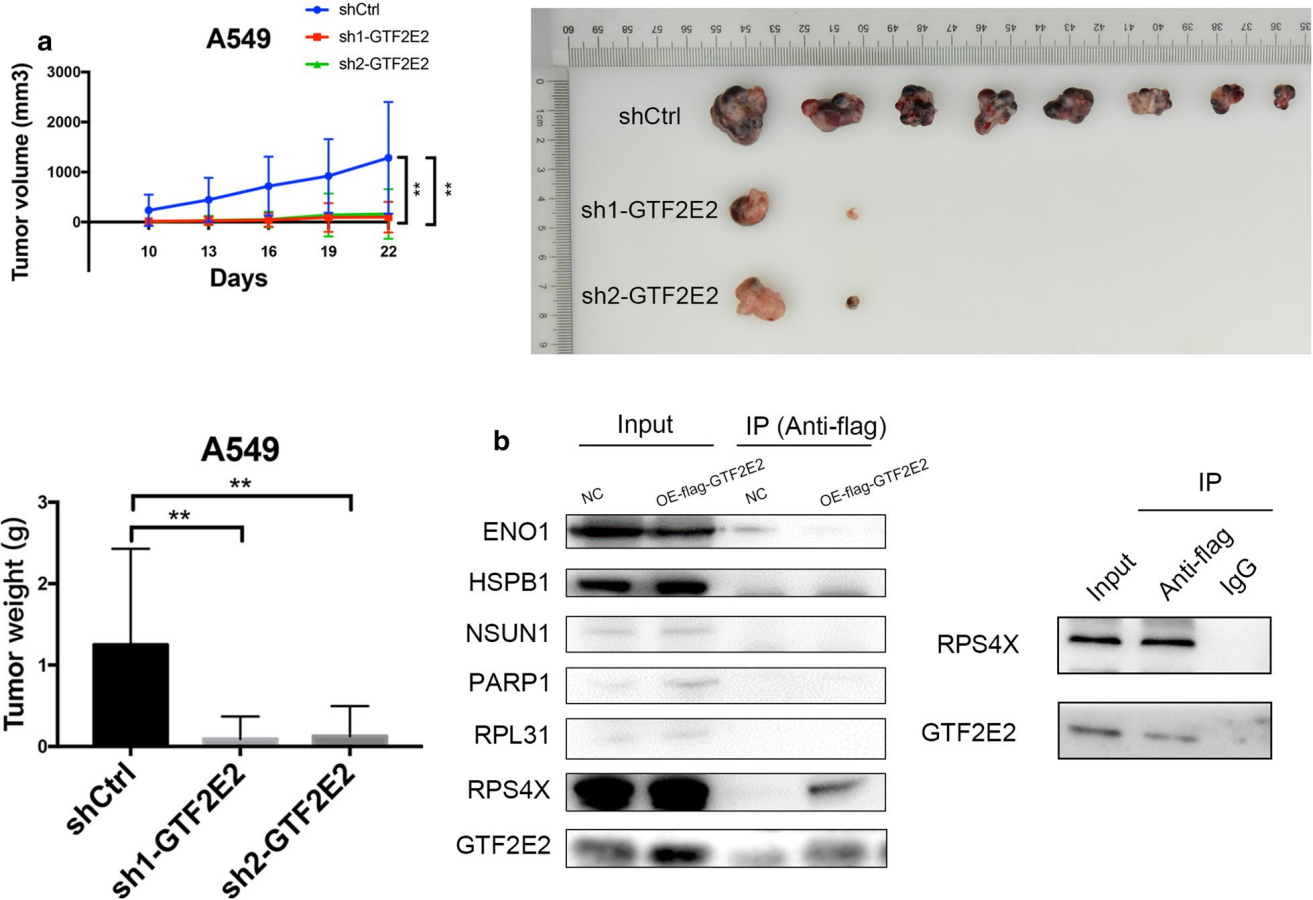
**a** The effects of GTF2E2 knockdown on tumor growth in mouse xenograft model. **b** Western blotting verifying the results from co-immunoprecipitation. IgG was used as negative control. **p < 0.01

Next, to determine whether GTF2E2 expression had any effect on tumor growth in vivo, we performed a tumor formation assay by subcutaneously injecting GTF2E2-underexpressed A549 cells or control cells into the flanks of nude mice. We found that GTF2E2 knockdown by shRNA significantly inhibited tumor growth in the mouse model: the knockdown of GTF2E2 lead to complete disappearance of the tumor in 6 out of 8 mice and a significant shrinkage in the other two mice, which was consistent with our in vitro results (Fig. 4a). Together, our in vitro and in vivo data revealed a critical role of GTF2E2 in the maintenance of malignancy in LUAD cells.

### GTF2E2’s potential interaction with RPS4X

To investigate how GTF2E2 regulates cellular signaling effectors to confer the increased malignancy of LUAD, we examined potential GTF2E2 interacting proteins in GTF2E2 overexpressing cells and corresponding control by shotgun liquid chromatography coupled with tandem mass spectrometry (LC–MS/MS) analyses. The identified 62 proteins that potentially interacted with GTF2E2 were listed in Additional file 1: Table S2. Next, we tried to validate these results by CO-IP. We found that only the interacting relationship between GTF2E2 and oncogenic ribosomal protein S4 X-linked (RPS4X), which encodes S4, a component of the 40S ribosomal subunit, was repeatable. After overexpressing flag-tagged GTF2E2 and flag-tagged RPS4X, as well as corresponding negative control vector, in 293T cells respectively, endogenous RPS4X was co-immunoprecipitated by flag antibody in flag-GTF2E2 OE cells, and the endogenous GTF2E2 was also reciprocally co-immunoprecipitated in flag-RPS4X OE cells. However, we failed to observe the co-immunoprecipitation of other LS–MS/MS identified proteins by flag antibody (Fig. 4b). Therefore, we chose RPS4X as the potential downstream interacting factor of GTF2E2 for subsequent analysis.

### GTF2E2 activates RPS4X to promote LUAD development through mTOR pathway

**Fig. 5:**
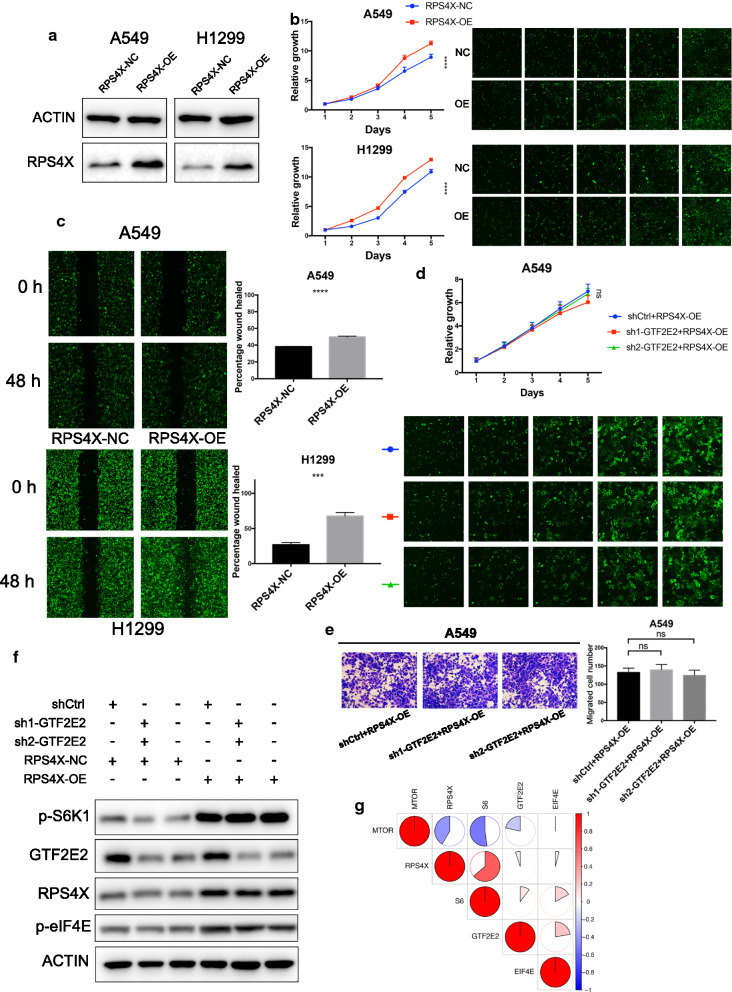
**a** Western blotting analyses verifying the RPS4X overexpressing efficiency in A549 and H1299 cells. **b**, **c** The effects of RPS4X overexpressing on cell proliferation (**b**), and migration (**c**) in A549 and H1299. **d**–**e** The effects of GTF2E2 knockdown on cell proliferation (**d**) and invasion (**e**) in RPS4X overexpressing A549 cells. **f** Western blotting analyses exhibiting the changes of proteins in mTOR signaling pathway after GTF2E2 knockdown in RPS4X overexpressing A549 cells. **g** The correlation matrix showing the correlations among GTF2E2, RPS4X, and mTOR pathway related proteins

As shown in Fig. 5a, the overexpression of RPS4X in both A549 and H1299 cell lines generated an opposite effect compared with GTF2E2 knockdown, leading to increased tumor cell proliferation and migration as well as tumorigenesis (Fig. 5b, c). This finding provides a potential link between GTF2E2 and RPS4X in the development and progression of LUAD cells. To determine whether GTF2E2 signals through RPS4X to mediate cancer cell proliferation, we further knocking down GTF2E2 in RPS4X-OE-A549 cells. As shown in Fig. 5d, e, when upregulating RPS4X, the alteration of GTF2E2 expression level could not generate a significant impact on A549 cells proliferation and migration anymore, indicating that in this condition, GTF2E2’s activating effect on RPS4X will not further enhance since the expression of PRS4X has reached a considerable level. Taken together, we inferred that GTF2E2 led to tumor progression via the interaction with RPS4X.

RPS4X is involved in various biological pathways in cancer cells via direct or indirect interaction with other genes [18–21]. Especially, Zhou et al. has proved the regulatory function of RPS4X on hepatocellular carcinoma’s tumorigenesis and metastasis by promoting the phosphorylation of S6K1 and eIF4E, which is indicative of the activation of mTOR signaling pathway and associated with mTOR-induced protein synthesis [21]. Considering the critical role that mTOR signaling pathway plays in multiple tumor types [22, 23], this novel discovery prompted us to wonder whether a similar mechanism exists in LUAD when RPS4X is activated by upstream molecules, like GTF2E2. Therefore, we investigated the phosphorylation level of S6K1 and eIF4E when altering the expression level of GTF2E2 and RPS4X separately and simultaneously. As shown in Fig. 5f, knocking down GTF2E2 attenuated S6K1 and eIF4E phosphorylation, while overexpressing RPS4X in GTF2E2-KD A549 cells significantly rescued this phenomenon. Besides, we also find a strong correlation between the expression level of RPS4X and S6K1, GTF2E2 and EIF4E, as well as mTOR and RPS4X or GTF2E2 in TCGA database (Fig. 5g), further demonstrating the potential complicated association among these factors. Therefore, based on above results, it is reasonable to conclude that GTF2E2 activates RPS4X by direct interaction, thus leading to LUAD development through mTOR pathway.

## Discussion

The abnormal expression of oncogenes or tumor suppressors plays a vital role in tumorigenesis and progression. Currently, although surgical resection combined with adjuvant chemotherapy or radiotherapy remains the primary treatment for LUAD, numerous new therapies directed against defined molecular targets have been emerging during the past few years [7], emphasizing the importance of screening new therapeutical targets. In this study, we provide a comprehensive view of GTF2E2’s potential function in the development of LUAD. We found that GTF2E2 expression was significantly upregulated in advanced tumor tissues of LUAD compared with that in early tumor or adjacent non-tumor tissues. The robustness of this result could be verified by the application of multiple bioinformatical and experimental approaches. Meanwhile, the expression level of GTF2E2 is negatively associated with LUAD patients’ overall survival. Both in vitro and in vivo assays showed that knockdown of GTF2E2 significantly inhibited the growth of LUAD cell lines. This phenomenon might be associated with the activation of RPS4X and consequently enhanced phosphorylation of eIF4E and S6K1, which are both downstream factors of mTOR signaling pathway. Together, our study demonstrated that GTF2E2 might function as an oncogene and negative prognostic factor in LUAD, thus potentially providing new therapeutic targets for the patients suffering from this disease.

As a critical factor for RNA transcription initiation, previous research on GTF2E2 has been focusing on its involvement in trichothiodystrophy [24]. However, little is known about its function in tumorigenesis and tumor development. Supporting the high-throughput result from the Depmap database, it has been reported by Yang et al. in their bioinformatical analysis that GTF2E2 was associated with glioblastoma pathogenesis and prognosis by upregulating CDC20, thus participating in the regulation of cell cycle and p53 signaling pathway [12]. For the first time, our study reveals the oncogenic role of GTF2E2 in LUAD through both in vitro and in vivo assays. However, in the tumor formation assay, we noticed an interesting phenomenon that after knocking down GTF2E2, hardly could the subcutaneously injected tumor cells form a visible tumor in most of the mice, whereas in only one of the mice a quite large tumor, although still smaller than those in the group of shNC, was formed. Considering the results from in vitro assays, the GTF2E2 knocking down significantly inhibited the tumor cells’ ability to proliferate and form a tumor in vivo, explaining the disappearance or shrinkage of the tumors in this cohort. Besides, the results of in vivo assays are impacted by numerous factors like the animal’s genotypic milieu, immune state, and health condition, thus accounting for the high heterogeneity and variation in this group. Future research confirming this in a larger cohort of animals is warranted to further explore the role of GTF2E2 not only in LUAD but also in other tumor types.

To decipher the mechanism by which GTF2E2 regulates LUAD’s development, using CO-IP and IC-MS/MS analyses, we identified RPS4X, whose potential function in tumor development had been widely reported previously, as an essential factor in GTF2E2-mediated tumor progression. As a critical factor involved in ribosome assembling and mRNA catabolic process, RPS4X has been reported by Lebel et al. that its depletion confers cisplatin resistance but reduces the proliferative growth rate by interacting with Y-box binding protein 1 (YB-1) in breast and ovarian cancer cell lines [18, 20]. Meanwhile, Zhou et al. suggested that RPS4X served as the downstream factor of SLFN11 and exhibited oncogenic function in hepatocellular carcinoma tumorigenesis and metastasis [21]. Besides, the prognostic value of RPS4X has also been reported in several cancer types [18–20]. These results were consistent with our findings in LUAD, indicating the critical role of RPS4X in the development of different tumor types. Considering the interaction between GTF2E2 and RPS4X, as well as their biological function, although the specific mechanism remains unclear, it is conceivable to infer that GTF2E2 promotes LUAD development by activating RPS4X, thus stimulating downstream biological pathways.

Inspired by Zhou et al.’s research in hepatocellular carcinoma, our study further demonstrated enhanced phosphorylation of S6K1 and eIF4E induced by RPS4X activation, indicating the participation of the mTOR signaling pathway in this regulatory axis. It has been previously confirmed that the PI3K/Akt/mTOR pathway stimulates tumor cell growth and proliferation by promoting a variety of anabolic processes in multiple cancer types [22, 25, 26], while the anti-cancer potential of mTOR inhibitors, like rapamycin and everolimus, has been clinically evaluated in lung cancer patients [27–29]. The mTOR function is mainly mediated by two downstream effectors, eIF4E and S6K1, which initiate eIF4E- and cap-dependent mRNAs translation, respectively, thus promoting tumorigenesis. Meanwhile, supporting our results, it has been widely reported that the phosphorylation of these two factors, caused by not only the mTOR pathway but also other molecules like MNK2, is tightly associated with NSCLC progression and proliferation thus indicating metastasis and unfavorable prognosis [30–33]. Additionally, we also noticed both negative and positive correlation among GTF2E2, RPS4X, and the factors mentioned above involved in the mTOR signaling pathway. This interesting phenomenon might be explained by the multiple bi-directional feedback loops occurring in mTOR signaling regulation, such as the best-characterized one: the S6K1-mediated negative feedback on pI3K [22]. Taken together, our findings implied that mTOR signaling molecules S6K1 and eIF4E might serve as the downstream effectors in GTF2E2-RPS4X induced LUAD development. Targeting these molecules may guide a more precise and personalized therapeutic strategy for patients suffering from LUAD. However, considering the preclinical nature of this study, further prospective and clinical exploration focusing on this mechanism is still warranted.

## Conclusion

In this study, we determined that GTF2E2 was upregulated in LUAD and was significantly associated with worse overall survival in LUAD patients. Functional assays and bioinformatic analyses revealed that GTF2E2 regulated LUAD cells’ growth by activating RPS4X, and the mTOR pathway appears to be a potential downstream pathway of this axis. Overall, GTF2E2 might serve as a promising biomarker for the diagnosis and prognosis of LUAD patients, thus shedding light on the personalized therapeutic regimen for LUAD in the future.

## Supplementary Information

**Additional file 1:** **Table S1.** The sequences of all the shRNAs and primers used in this study. **Table S2.** Results from shotgun liquid chromatography coupled with tandem mass spectrometry analyses.

## Data Availability

All data generated or analyzed during this study are submitted to the journal.
